# Supplementary material for: Learning curve and surgical outcome of robotic assisted colorectal surgery with ERAS program
Source: Sci Rep. 2022 Nov 29;12:20566. doi: 10.1038/s41598-022-24665-w (PMC9709162; doi:10.1038/s41598-022-24665-w)
Supplement: Supplementary file 1 — Supplementary Information 1. [file 41598_2022_24665_MOESM1_ESM.zip › LSC Raw data-Ñ[▒K/050/OP_ñw╝╨░O▒Kñσ.pdf]

正式摘要(電子簽章：0000001783295554)

W95 / W95

臺中榮民總醫院

Operation Note

[redacted]

- 手術日期：2019/10/16
- 手術醫師：VS 林俊余 , CR 江梓維 , CR 黃偲霖 , R 陳映菁 , R 林陳彥 , R 林宜慧
- Anesthesia : GE
- Position : Lithotomy
- Aseptic agents : Soap , Betadine Aqua

Diagnosis

- Pre-operative diagnosis : rectum ca 2 CM FAV
- Post-operative diagnosis : rectum ca 2 CM FAV s/p lapa LAR ISR Rt LLND ilesotmy, sT2N0M0

Surgical Indication

- Removal of rectal tumor and pathological examination

Operation

- laparoscopic Low anterior resection for neogrowth of colorectum

---

Finding

- 1. a scarr 1*1 cm in size 1 cm above dnetal line , sTxN0M0
  2. LN(-) liver metastasis(-)
  3. margin: distal 0.5cm, proximal 15cm
  4. Rt LLND was done. ascites(-)
  5. penrose in pelvis.

Procedure

- . Under GE, the patient was put in lithotomy position. 5 troca hole was made, the first was by open method. Pneumoperitonium was created. the Olympus Endo-EYE laparoscope was inserted from umbilical port. Abdominal exploration was done and the findings were mentioned above. The patient position was adjusted to head-down and right tilting to let small bowels move to RUQ and the pelvis was well exposured. The IMA was identified, dissected, skeletonized by Harmonic scalpel and transected by triple clips. The IMV was done in the same way. The mobilization of S-colon was done with dissection via embryonic plane between Gerota fascia and meso-sigmoid colon. The lateral attachement of sigmoid colon was also divided. After fully mobilization of S-colon, the rectum was also dissected deep to rectococcygeal ligament posteriorly, Denovillier fascia anteriorly.
  Then enter ISR phase, open anus with long star. found internal sphincter and resect in ISR plain. Transection of rectum at peritoneal reflection was done . The speciemn was removed from anus. The end to end colorectal anastomosis was done by hand sew method. penrose in pelvs. 5 ports and was closed in layers. ilestyomy was made in RLQ. Then do the Rt LLND smoothly after seperarte ureter. Throughout the whole course of operation, patient stood quite well.
- Estimated blood loss: minimum
- 器械數量: 正確
- 紗布數量: 正確

Image

- PAINT
  - colon & rectum

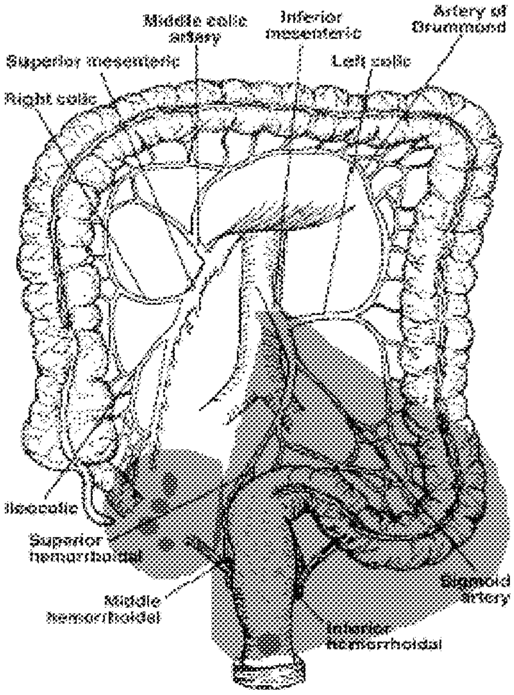

編輯主治 2222H林俊余

正式摘要**(電子簽章：000000178329554)**

W95 / W95

doctorRank:B
